# Integrated biomarker profiling of the metabolome associated with type 2 diabetes mellitus among Tibetan in China

**DOI:** 10.1186/s13098-023-01124-8

**Published:** 2023-07-01

**Authors:** Jinli Meng, Fangfang Huang, Jing Shi, Chenghui Zhang, Li Feng, Suyuan Wang, Hengyan Li, Yongyue Guo, Xin Hu, Xiaomei Li, Wanlin He, Jian Cheng, Yunhong Wu

**Affiliations:** 1 Department of Radiology, Hospital of Chengdu Office of People’s Government of Tibetan Autonomous Region (Hospital. C.T.), No. 20, Xi Mian Qiao Heng Jie, Wuhou District, Chengdu, Sichuan China; 2 Hubei University of Chinese Medicine, Wuhan, 430065 China; 3 Department of Science and Education Section, Hospital of Chengdu Office of People’s Government of Tibetan Autonomous Region (Hospital. C.T.), No. 20, Xi Mian Qiao Heng Jie, Wuhou District, Chengdu, Sichuan China; 4 Department of Endocrinology and Metabolism, Hospital of Chengdu Office of People’s Government of Tibetan Autonomous Region (Hospital. C.T.), No. 20, Xi Mian Qiao Heng Jie, Wuhou District, Chengdu, Sichuan China; 5 Department of Neurosurgery, West China Hospital, Sichuan University, Chengdu, China

**Keywords:** Tibetan, Type 2 diabetes mellitus, Serum metabolomics, Machine learning, Biomarker

## Abstract

**Introduction:**

Metabolomic signatures of type 2 diabetes mellitus (T2DM) in Tibetan Chinese population, a group with high diabetes burden, remain largely unclear. Identifying the serum metabolite profile of Tibetan T2DM (T-T2DM) individuals may provide novel insights into early T2DM diagnosis and intervention.

**Methods:**

Hence, we conducted untargeted metabolomics analysis of plasma samples from a retrospective cohort study with 100 healthy controls and 100 T-T2DM patients by using liquid chromatography–mass spectrometry.

**Results:**

The T-T2DM group had significant metabolic alterations that are distinct from known diabetes risk indicators, such as body mass index, fasting plasma glucose, and glycosylated hemoglobin levels. The optimal metabolite panels for predicting T-T2DM were selected using a tenfold cross-validation random forest classification model. Compared with the clinical features, the metabolite prediction model provided a better predictive value. We also analyzed the correlation of metabolites with clinical indices and found 10 metabolites that were independently predictive of T-T2DM.

**Conclusion:**

By using the metabolites identified in this study, we may provide stable and accurate biomarkers for early T-T2DM warning and diagnosis. Our study also provides a rich and open-access data resource for optimizing T-T2DM management.

**Supplementary Information:**

The online version contains supplementary material available at 10.1186/s13098-023-01124-8.

## Introduction

Diabetes mellitus (DM) is a common chronic metabolic disease characterized by hyperglycemia resulting from insulin-omission [1]. Type 2 diabetes mellitus (T2DM), which accounts for more than 95% of all DM cases [2], is an important cause of diabetic complications and the high mortality in individuals with DM [3, 4]. Currently, approximately 462 million individuals suffer from T2DM worldwide, and China obtained roughly 24.4% (102.9 million) of all cases [5]. Recent surveys in China estimated that the overall prevalence of DM is 10.9%, and that of pre-DM is 35.7%. Among Tibetans, the age-standardized prevalence of DM and pre-DM was 6.2% and 19.7%, respectively, and continues to increase rapidly [6, 7].

The Tibetan Chinese population is one of the two largest human plateau-dwelling groups globally [8]. The risk factors of DM and the characteristics of glucose metabolism in Tibetans have already been extensively reported. For example, DM among Tibetans is associated with a higher annual family income, alcohol consumption, and higher fasting plasma glucose (FPG) level, independent of age, sex, and body mass index (BMI) [9]. Tibetans tend to reduce fatty acid oxidation and increase glycolysis to decrease tissue oxygen demand, resulting in lower FPG levels and higher lactate and free fatty acid concentrations [10]. Currently, reports regarding T2DM characteristics among Tibetans in China remain limited.

Early detection, diagnosis, and treatment of T2DM are challenging. Metabolomics identifies changes in the metabolic profile and particular metabolic abnormalities, thereby a powerful technique for studying disease-relevant metabolic processes and dysregulation. Theoretically, liquid chromatography mass spectrometry (LC-MS) is the most common and ideal profiling technology used for detecting serum biomarkers [11]. Several prospective metabolomic investigations have identified numerous novel metabolites predictive of T2DM risk, including branched-chain amino acids (BCAAs) (e.g., leucine, isoleucine and valine) [12, 13], aromatic amino acids (phenylalanine and tyrosine) [13–15], other amino acids, acylcarnitines and certain lipids [16]. However, metabolomic signatures of incident DM among Tibetans remain largely unclear, and effective and reliable biomarkers for early T-T2DM diagnosis remain unknown. Considering the Tibetans’ particular glucose metabolism and genetic determinants of Tibetan high-altitude adaptation, evidence for the association of Tibetan T2DM (T-T2DM) with other amino acids or other types of metabolites is still very limited. Furthermore, insight into whether ethnic differences in these metabolite concentrations potentially contribute to the higher risk of T2DM remains uncertain. Therefore, we need to determine the metabolite concentrations, identify the associations with T-T2DM, and seek to establish ideal biomarkers for the early and accurate diagnosis of T-T2DM.

In the present study, we aimed to perform a metabolome-wide analysis of T2DM among Tibetans adults. We sought to reveal the clinical characteristics and metabolite signatures associated with T-T2DM. We found phenylalanine metabolism, phenylalanine, tyrosine and tryptophan biosynthesis, arachidonic acid metabolism as key disturbed pathways in T-T2DM. By employing machine learning and correlation analysis, we identified ten unique biomarkers and evaluated their diagnostic values for T-T2DM.

## Research design and methods

### Study design and population

In the current study, we recruited 100 patients from Hospital of Chengdu Office of People’s Government of Tibetan Autonomous Region (Hospital. C.T.) Sichuan, China, between 31 and 2020 and 30 October 2021. Our study was conducted in accordance with the Declaration of Helsinki and approved by the Institutional Review Board for Clinical Research of Hospital. C.T. A comprehensive battery of surveys and a clinical assessment with fasting blood draw were conducted by trained, certified, and bilingual staff at in-person clinic visits from October 2020 to October 2021. The study was approved by the institutional review boards at all participating institutions, and all participants gave written informed consent. *Inclusion criteria* include (1) 20–75 years old; (2) diagnostic criteria of 2-DM (an fasting plasma glucose (Glu0) ≧ 7.0mmol/l and/or a 2-h blood glucose (Glu120) level ≧ 11.1mmol/l and/or an haemoglobin A1c (HbA1c) ≧ 6.5%, all patients); (3) Blood pressure below 140/90 mmHg; and (4) signed informed consent. *Exclusion criteria* include (1)T1DM or other specific types of diabetes mellitus; (2) acute complications of DM; (3) complication of serious primary diseases in cardiovascular, cerebrovascular, liver, kidney, and the hematopoietic system as well as a tumor; (4) suffering from mental illness and unable to cooperate; (5) pregnant or lactating women, or those preparing for pregnancy, women in their menstrual period; (6) recent use of psychoactive drugs or hormones; and (7) those who have participated in other clinical trials within the past 1 month.

**Fig. 1:**
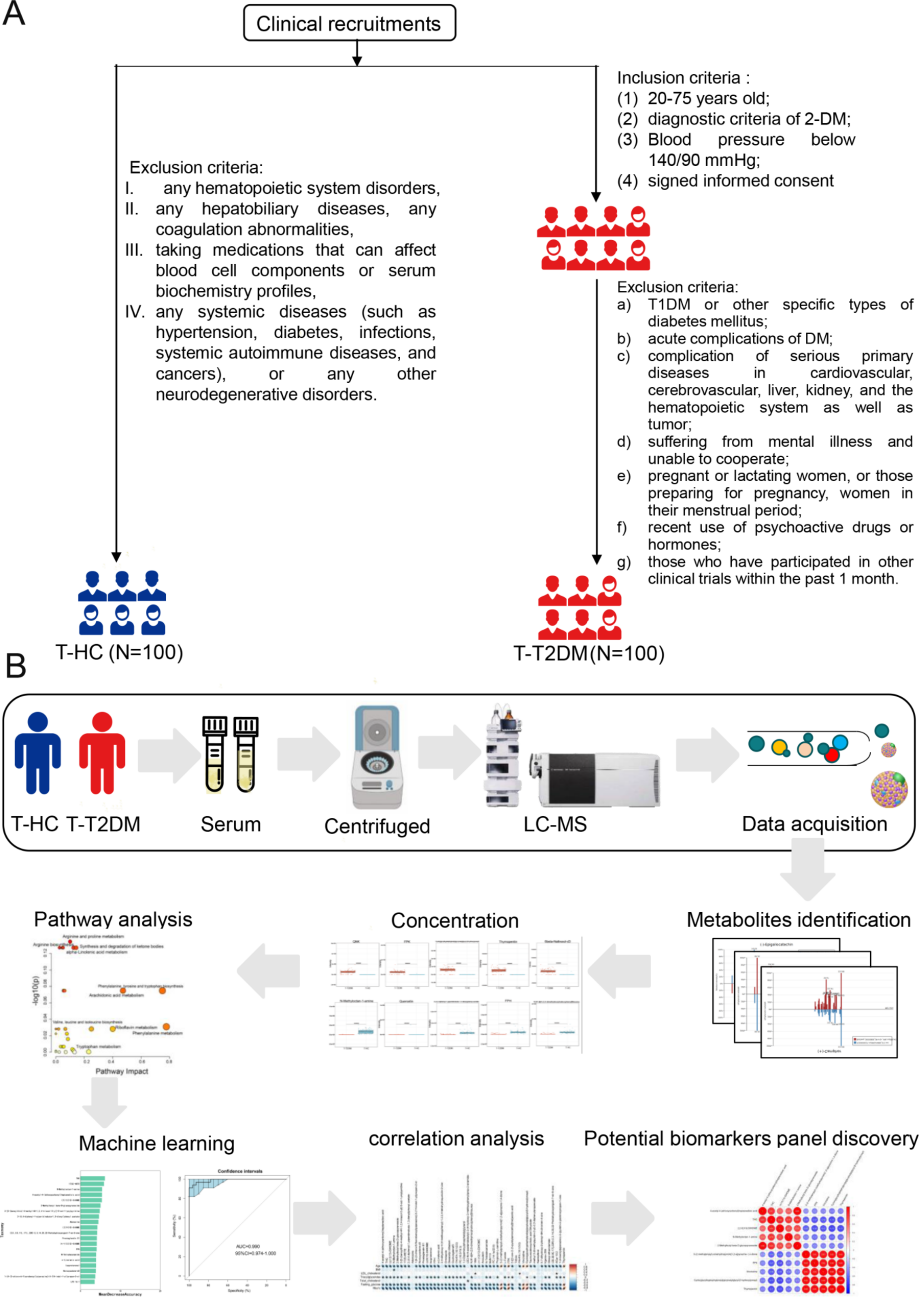
Study design. **(A)** The flowchart of patients enrolled in this study. T1DM, type 1 diabetes mellitus; DM, diabetes mellitus; T-T2DM, Tibetan type 2 diabetes mellitus; T-HC, Tibetan healthy controls. **(B)** Overview of workflow in this study. LC-MS, liquid chromatography-mass spectrometry

Healthy controls, who participated in yearly health screenings during the study period and had no clinical evidence of glaucoma or a family history of glaucoma, were also consecutively enrolled from Hospital. C.T. Exclusion criteria of healthy controls: any hematopoietic system disorders, any hepatobiliary diseases, any coagulation abnormalities, taking medications that can affect blood cell components or serum biochemistry profiles, any systemic diseases (such as hypertension, diabetes, infections, systemic autoimmune diseases, and cancers), or any other neurodegenerative disorders. According to the inclusion and exclusion criteria, a total of 100 healthy controls were included (Fig. 1A**)**. Demographics and clinical parameters of patients with T2DM (n = 100) and healthy controls (n = 100) were shown in Table 1.

**Table 1:** Demographic and clinical characteristics of the enrolled patients with T2DM (N = 100) and health controls (N = 100)

Characteristics	T2DM (n = 100)	HC (n = 100)	P value	
Gender				
male, %	60 (60)	50 (50)	0.155	
female, %	40 (40)	50 (50)		
Age (years)	51.41 ± 9.05	40.44 ± 11.26	< 0.0001	
Current smoker, %	28 (28)	-	-	
Current drinker, %	23 (23)	-	-	
BMI (kg/m2)	25.84 ± 3.41	24.90 ± 3.83	0.069	
Waist circumference, cm	94.9 ± 9.38	-	-	
Systolic blood pressure (SBP)	119.21 ± 12.37		-	
Diastolic blood pressure (DBP) 77.83 ± 7.93		-	-	
LDL cholesterol (mmol/L)	2.63 ± 0.78	2.79 ± 0.70	0.123	
Total cholesterol (mmol/L)	4.47 ± 0.92	4.61 ± 0.80	0.264	
Triglycerides (mmol/L)	1.53 ± 0.76	1.31 ± 0.75	0.038	
Serum uric Acid (mg/dL)	331.51 ± 69.44	369.02 ± 93.32	0.001	
Serum Creatinine (µmol/L)	59.19 ± 12.45	64.55 ± 10.15	0.001	
ALT (U/L)	33.09 ± 21.04	-	-	
AST (U/L)	20.69 ± 11.67	-	-	
GGT (U/L)	59.06 ± 54.91		-	
Fasting glucose (mmol/L)	7.90 ± 2.08	5.24 ± 2.46	< 0.0001	
2 h OGTT (mmol/L)	12.17 ± 3.66	-	-	
HbA1c (%)	10.33 ± 2.47	5.91 ± 1.07	< 0.0001	

Note: values are presented as the mean ± SD or n (%). T2DM, type 2 diabetes mellitus. HC, health controls. BMI, Body Mass Index. AST, aspartate aminotransferase, ALT, alanine aminotransferase, GGT, gamma- glutamyl transpeptidase. OGTT, oral glucose tolerance test. HbA1c, glycated haemoglobin

Figure 1B provides a detailed workflow of the metabolomics study. First, we collected 200 serum samples from the two groups and used metabolomics to identify the metabolite biomarkers of T-T2DM. We then used the metabolites identified by differential expression analyses to test machine learning models. This analysis highlighted potential biomarkers for clinical diagnosis and pathways involved in T-T2DM onset and progression.

### Measurements of blood glucose and covariates

We collected information regarding gender, age, lifestyle factors, medical history, sociodemographic characteristics, and family history using a standardized questionnaire [17]. Systolic blood pressure (SBP) and diastolic blood pressure (DBP) and waist circumference were performed following standardized protocols [18]. Body mass index (BMI) was calculated as weight in kilograms divided by height in meters squared. Centralized laboratory tests were performed to determine plasma fasting glucose and hemoglobin A1c (HbA1c), and serum uric acid, creatinine, liver enzymes and lipids including triglycerides, and total, low-density lipoprotein (LDL), and high-density lipoprotein (HDL) cholesterol. For T-T2DM participants, 2-h plasma glucose levels were also measured following a standard 75-g 2-h oral glucose tolerance test (OGTT).

### Sample collection

Participants were asked to fast for at least 8 h before the examination, consume only water and necessary medications, and to refrain from smoking or physical activity before undergoing the fasting examination procedures. Venous blood samples were collected, processed, and frozen (at -80 °C) on-site toward the beginning of the visit.

### Serum metabolomics profiling by liquid chromatography mass spectrometry (LC-MS) (LC-MS)

The prepared samples were analyzed using an ultraperformance HPLC (UHPLC) system (1290, Agilent Technologies) with a UPLC HSS T3 column (2.1 mm × 100 mm, 1.8 mm, Waters) coupled to Q Exactive Focus (Thermo Fisher Scientific, MA, USA), via a previously described method with some modifications [19]. Additional details are provided in the **Supplementary Material Methods** section.

### Machine learning prediction on metabolomics data

For each pairwise comparison, based on the features selected by the differential metabolite analysis, random forest classification was applied by R and Bioconductor packages ‘random forest’ and ‘ggplot2’. The random forest classification (RFC) for identifying T-T2DM was trained on 140 randomly selected subjects (67 healthy subjects, 73 with T-T2DM), and then tested on the remaining subjects (33 healthy subjects, 27 with T-T2DM). The analysis was conducted with 5 repetitions of 10-fold cross-validation, using cross-validation error curves to select features as described elsewhere [20]. To improve the sensitivity of the integrated biomarker profiling (IBP) prediction model for T-T2DM, analysis of variance, Mean Decrease in Accuracy, and Gini impurity were used to rank potential biomarkers by importance [21]. The Receiver operating characteristic (ROC) curves and area under the curve (AUC) were calculated by R package ‘pROC’. The AUC, accuracy, sensitivity, specificity, and precision were used to evaluate the model performance. Detailed methods are in the **Supplementary Material Methods **section.

### Data analysis

The clinical characteristics of patients were compared using the Fisher’s exact test for categorical variables and the Wilcoxon rank-sum test for continuous variables. These metabolites were annotated using the KEGG database (https://www.genome.jp/kegg/pathway.html), Human Metabolome Database (HMDB) (https://hmdb.ca/ metabolites) and LIPID MAPS Structure Database (http://www.lipidmaps.org/). Principal components analysis (PCA) and Partial least squares discriminant analysis (PLS-DA) were performed at metaX [22]. We applied univariate analysis (t-test) to calculate the statistical significance (P-value). The metabolites with VIP > 1 and P-value < 0.05 and fold change ≥ 1.2 or FC ≤ 0.833 were considered to be differential metabolites. Clustering heat maps were plotted by Pheatmap package in R language. Volcano plots were performed by ggplot2 in R. The correlation between metabolites and clinical characteristics were analyzed by corrplot package in R (method = pearson), P-value < 0.05 was considered as statistically significant. The metabolic pathways were considered as enrichment by impact values, when P-value of metabolic pathway < 0.05, metabolic pathway was considered as statistically significant enrichment.

The variables were selected based on variable importance in the projection (VIP > 1.0) from the peak height. In addition to the multivariate statistical method, Student’s t-test was also applied to measure the significance of each lipid. The resultant p values for each metabolite in all cross-comparisons were corrected by the Bonferroni correction. The resultant P values from ANOVA were further adjusted by the false discovery rate (FDR) based on the Hochberg-Benjamini method. Significantly altered variables were defined and further identified by VIP > 1.0, P < 0.05, and FDR < 0.05.

### Statistics

All the statistical analyses were performed by statistical software R (R version R-3.4.3) with corresponding packages available. P-value ≤ 0.05 (or FDR = 5% for multiple hypothesis testing) was used to define significance.

## Result

### Cohort characteristics, sample collection

Table 1 enumerates the characteristics of the Tibetan participants. We included 100 patients with T-T2DM (40 females and 60 males) and 100 healthy controls (T-HC; 50 females and 50 males). The median (interquartile range, IQR) age of all participants was 47 (32–54) years. The T-T2DM cohort aged 32–65 years old, with a BMI of 18.20–38.97 kg/m^2^. Factors such as sex, BMI, low-density lipoprotein cholesterol level, and total cholesterol level were not significantly different between the T-T2DM and T-HC groups. The T-T2DM group was more likely to be hypercholesterolemic and had significantly higher triglyceride levels and lower serum uric acid and creatinine levels than the T-HC group. The mean values for FPG and 2-hour oral glucose tolerance test in the T-T2DM group were 7.90 ± 2.08 and 9.19 ± 1.16 mmol/L, respectively. Clearly, the T-T2DM group had higher levels of FPG and glycosylated hemoglobin (HbA1c) than the T-HC group.

### Individual metabolites and risk of diabetes

**Fig. 2:**
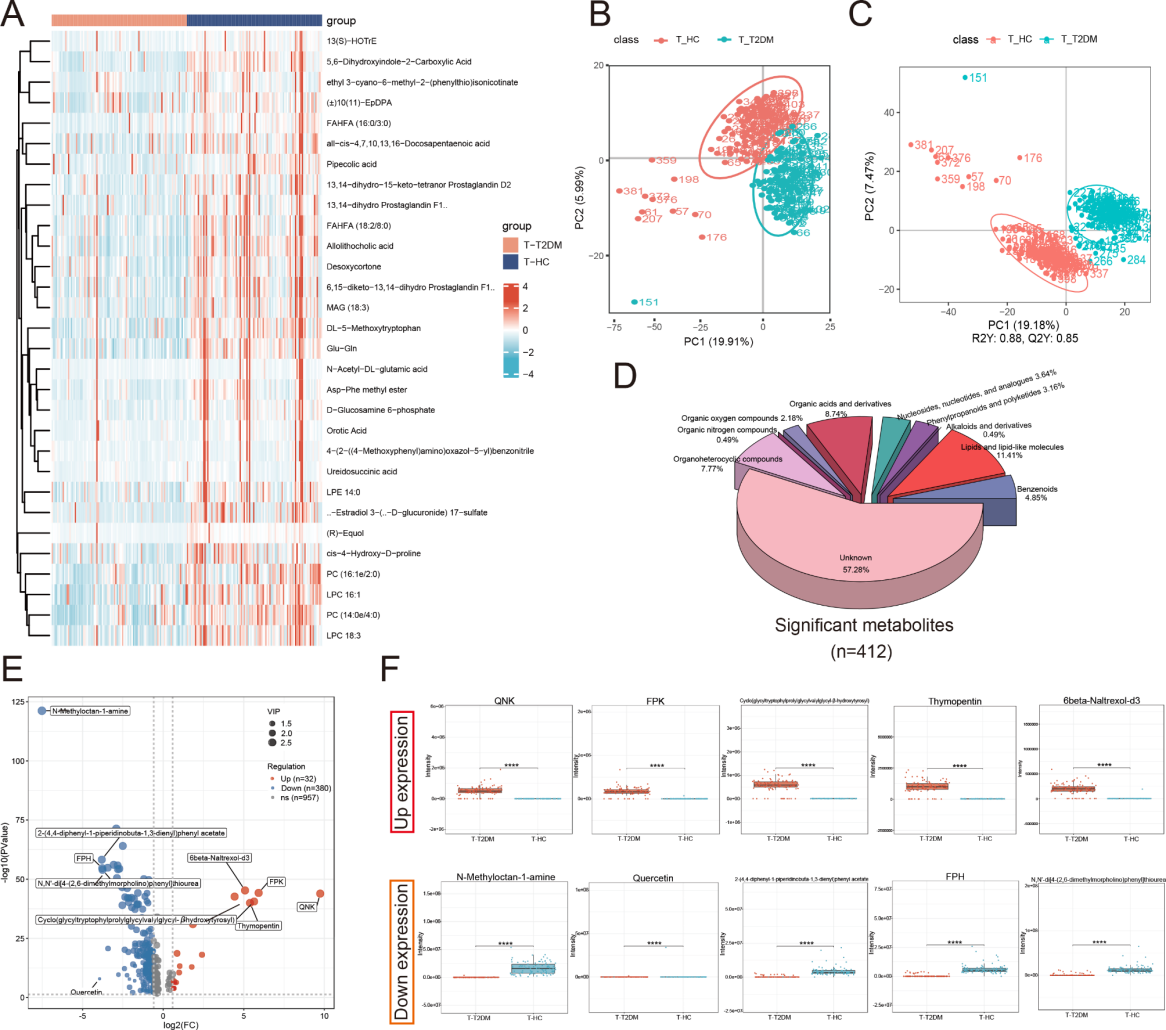
Detection of differentially expressed metabolites by pairwise comparison of T-T2DM. **(A)** Hierarchical clustering of differentially expressed metabolites. The median expression levels of metabolites (n = 30) for two groups are presented in the heatmap. **(B)** Score plots from the PCA model derived from the UPLC-MS profile of serum in two groups. **(C)** Score plots from the OPLS-DA model from metabolic profiles of two groups. **(D)** Pie-chart for the classification of significant differentially expressed metabolites (n = 412) according to meta-intensity. **(E)** Volcano plot of significantly differentially expressed metabolites with marking the top 5 up and down expression differential metabolites (Red represents up-regulated, blue represents down-regulated metabolites). **(F)** Representative box plots for top up-regulated and down-regulated metabolites

To identify the serum metabolome features of the patients in the T-T2DM and T-HC groups, we generated untargeted metabolome profiles from fasting serum samples by means of LC-MS. Figure 2 A shows the median expression of 30 differentially expressed metabolites in the two groups. Both the principal components analysis score plot (Fig. 2B**)** and orthogonal partial least squares discriminant analysis (OPLS-DA model: R2Y(cum) = 0.88, Q2Y(cum) = 0.85, Fig. 2C), which were validated by permutation tests (200 permutations), revealed significant metabolite differences between the T-T2DM and T-HC groups. Overall, 1369 metabolites have been detected in serum. Among them, 412 (30.09%) significantly correlated with incident T-T2DM. Furthermore, 236 of these 412 metabolites largely included lipids and lipid-like molecules (11.41%), organic acids and derivatives (8.74%), and organoheterocyclic compounds (7.77%), and 14.8% were within 6 other metabolites (Fig. 2D). Of these 412 significant metabolites, 32 were positively associated with DM risk, while the 380 remaining metabolites showed an inverse association (Fig. 2E). To identify differentially expressed metabolites by pairwise comparisons, we conducted a nonparametric Wilcox rank-sum test on each metabolite (Supplementary Tables 1 and Fig. 2F). The metabolites were mainly components of amino acid metabolism and lipid metabolism. The top 5 metabolites showing upregulated expression were glutamine-asparagine-lysine (QNK), phenylalanine-proline-lysine (FPK), cyclo (glycyltryptophylprolylglycylvalylglycyl-β-hydroxytyrosyl), thymopentin and 6beta-Naltrexol-d3. Conversely, N-methyloctan-1-amine, quercetin, 2-(4,4-diphenyl-1-piperidinobuta-1,3-dienyl) phenyl acetate, phenylalanine-proline-histidine (FPH) and N,N’-di[4-(2,6-dimethylmorpholino)phenyl]thiourea were the top 5 metabolites demonstrating downregulated expression.

### Correlation network of differential metabolites in serum

**Fig. 3:**
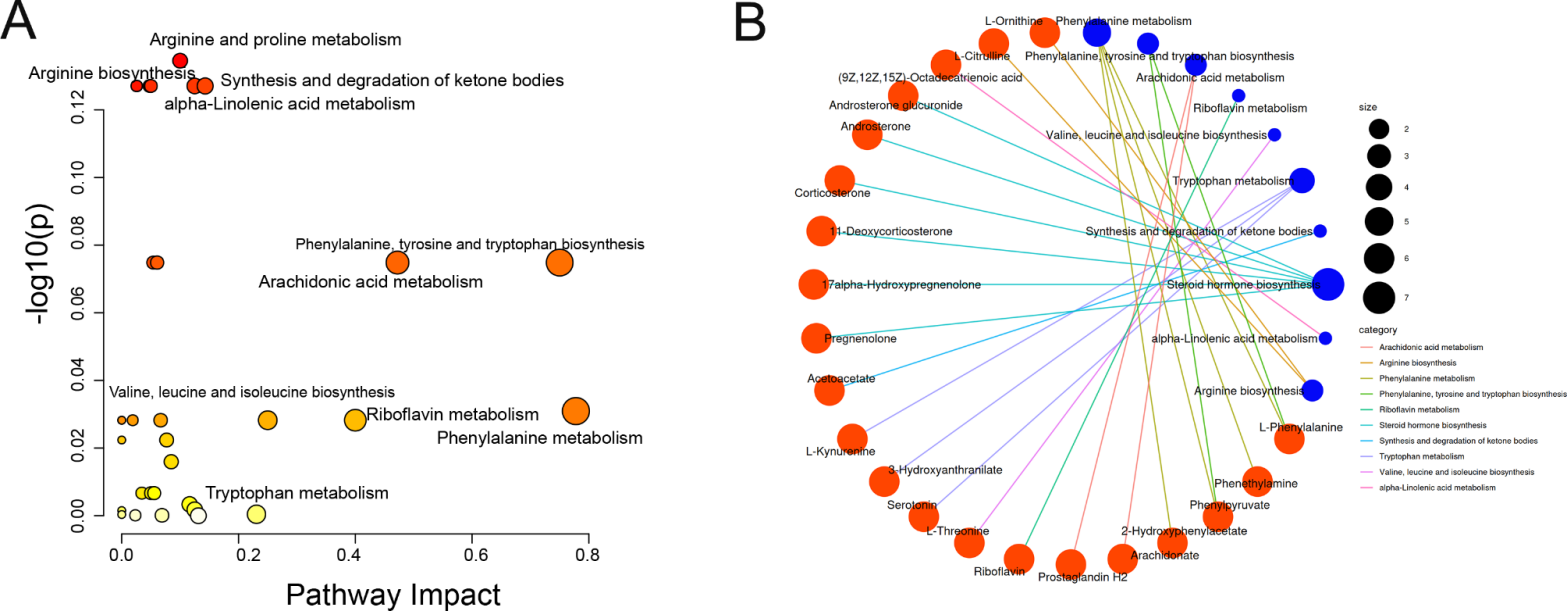
Correlation network of differential metabolites in serum. **(A)** KEGG analysis of significant functional pathways involved according to the differentially expressed metabolites. **(B)** Network analysis based on the top 10 KEGG pathways and their differential metabolites. The edges indicate the correlations between metabolites and metabolites, the size of node indicates the improtance of pathway (Red nodes represents metabolites, blue nodes represents pathways)

To explore the impact of metabolite alterations on T-T2DM, we conducted pathway and network analyses. As shown in Fig. 3A, we generated bubble plots to illustrate top significant pathways enriched by these biomarkers for each pairwise comparison. In the pathway analyses, phenylalanine metabolism; phenylalanine, tyrosine, and tryptophan biosynthesis; arachidonic acid metabolism, and riboflavin metabolism were superior. Consistently, our network analyses showed many key metabolites linked to T-T2DM development, including L-phenylalanine, phenylpyruvate, 2-hydroxyphenylacetate, arachidonate, and acetoacetate, and they were significantly altered in the T-T2DM and T-HC groups (Supplementary Tables 2 and Fig. 3B).

### Machine learning for the pairwise predictions of T-T2DM from serum metabolite expression

**Fig. 4:**
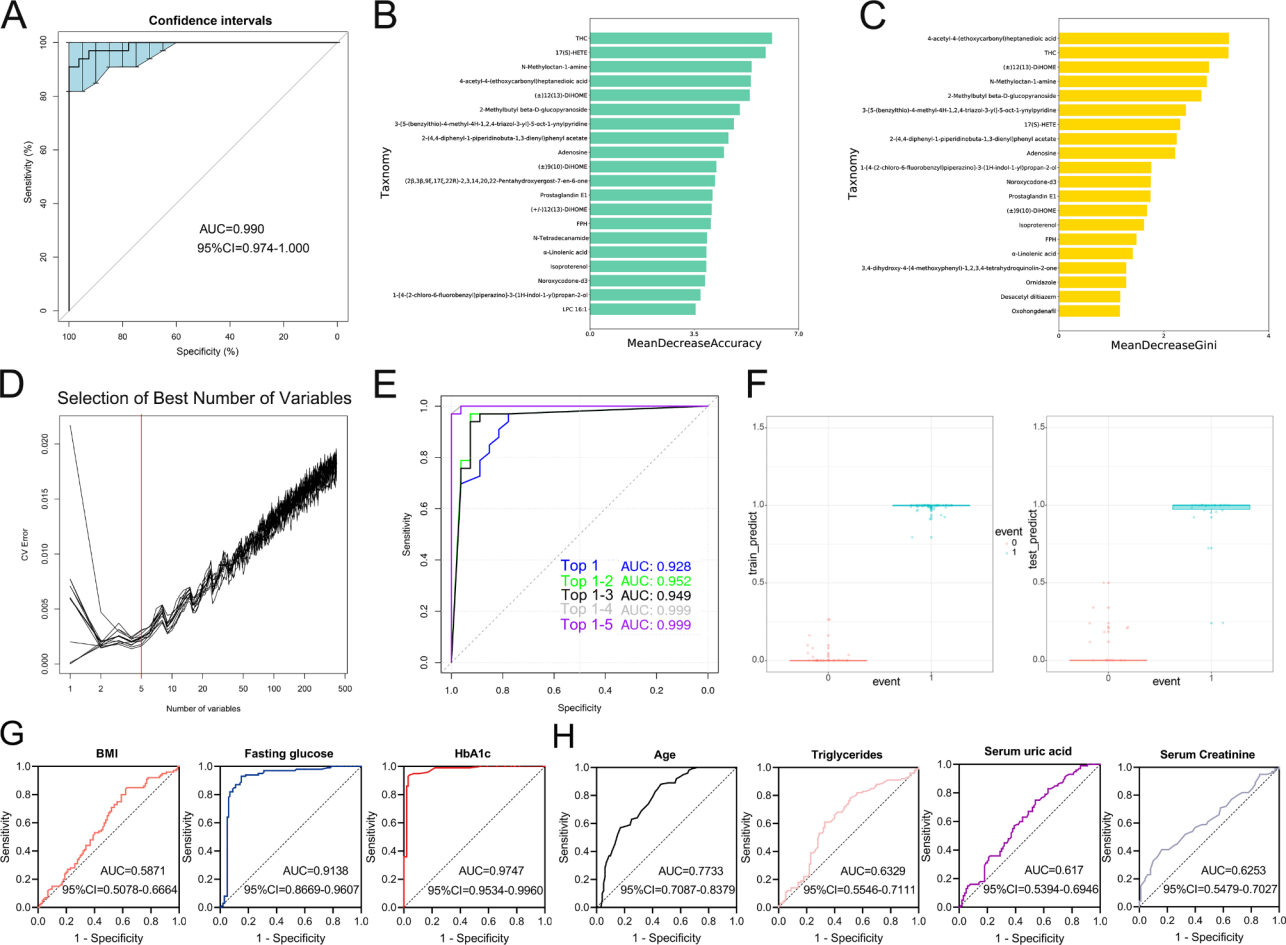
Establishment of integrated biomarker profiling. **(A)** AUC of the integrated 412 differential metabolites based on random forest classification (RFC) model. **(B)** the Mean Decrease in Accuracy (MDA) of 20 potential biomarkers. **(C)** Gini impurity of 20 potential biomarkers. **(D)** Distribution of 5 trials of 10-fold cross-validation error in random forest classifiers. The model was trained with 412 differential metabolites in the training set (T-T2DM group, n = 73; diabetes group, n = 67). The black solid curve showed the trials. The red line indicated the number of picked features in the optimal set. **(E)** AUC of the 5 selected potential biomarkers from the RFC model. **(F)** The prediction performance of the model consisted of 5 potential biomarkers in the train and test sets. **(G)** ROC curves for traditional markers BMI, fasting glucose and HbA1c. **(H)** ROC curves for risk factors of T-T2DM (age, triglycerides, serum uric acid and creatinine)

For verifying the values of the identified metabolites in predicting T-T2DM status, we established the random forest classification (RFC) model to investigate whether metabolic profiling could predict DM development in Tibetans, independent of the primary diagnostic criteria of DM (Glu0, Glu120, and HbA1c). Initially, the predictive performance of metabolites for T-T2DM prediction was examined using randomly selected participants classified into the training (n = 140) and validation datasets (n = 60). The area under the receiver operating characteristics (ROC) curve (AUC) was 99.0% (95% CI: 97.4–100%, Fig. 4A). Then, we assessed the individual contribution of each feature to the classification accuracy via the random forest variable importance analysis for each class in the two models by the Mean Decrease in Accuracy and Gini impurity, which indicates the importance of the feature for the classification performance. Figure 4B-C illustrates the relative importance of the 20 most predictive metabolites. The top 5 predictive metabolites were 4-acetyl-4-(ethoxycarbonyl)heptanedioic acid, threonine-histidine-cysteine (THC), (±)12(13)-DiHOME, N-methyloctan-1-amine, and 2-methylbutyl beta-D-glucopyranoside by Gini importance. Supplementary Fig. 1 shows the paired differences in metabolite concentration between two groups. Moreover, we generated ROC curves from a fivefold cross-validation for all metabolites and successfully constructed a model containing the five most predictive metabolites (Fig. 4D). The model exhibited an AUC of 99.9% for T-T2DM prediction (Fig. 4E**)**. Its prediction performance was estimated, and the T-T2DM group in the training and validation sets could be more broadly separated than the T-HC (Fig. 4F**)**. group. We also calculated the AUCs of the traditional diagnostic metrics (BMI, fasting glucose and HbA1c, AUC = 0.5871, 95%CI = 0.5078–0.6664, AUC = 0.9138, 95%CI = 0.8669–0.9607, AUC = 0.9747, 95%CI = 0.9534–0.9960, respectively, Fig. 4G) and compared the prediction performance of T-T2DM risk factors such as age, triglycerides, serum uric acid, and creatinine (Fig. 4H**).** Together, these results indicated that the metabolite prediction model provided a better predictive value than the clinical features.

### Potential biomarker panel discovery for predicting T-T2DM

**Fig. 5:**
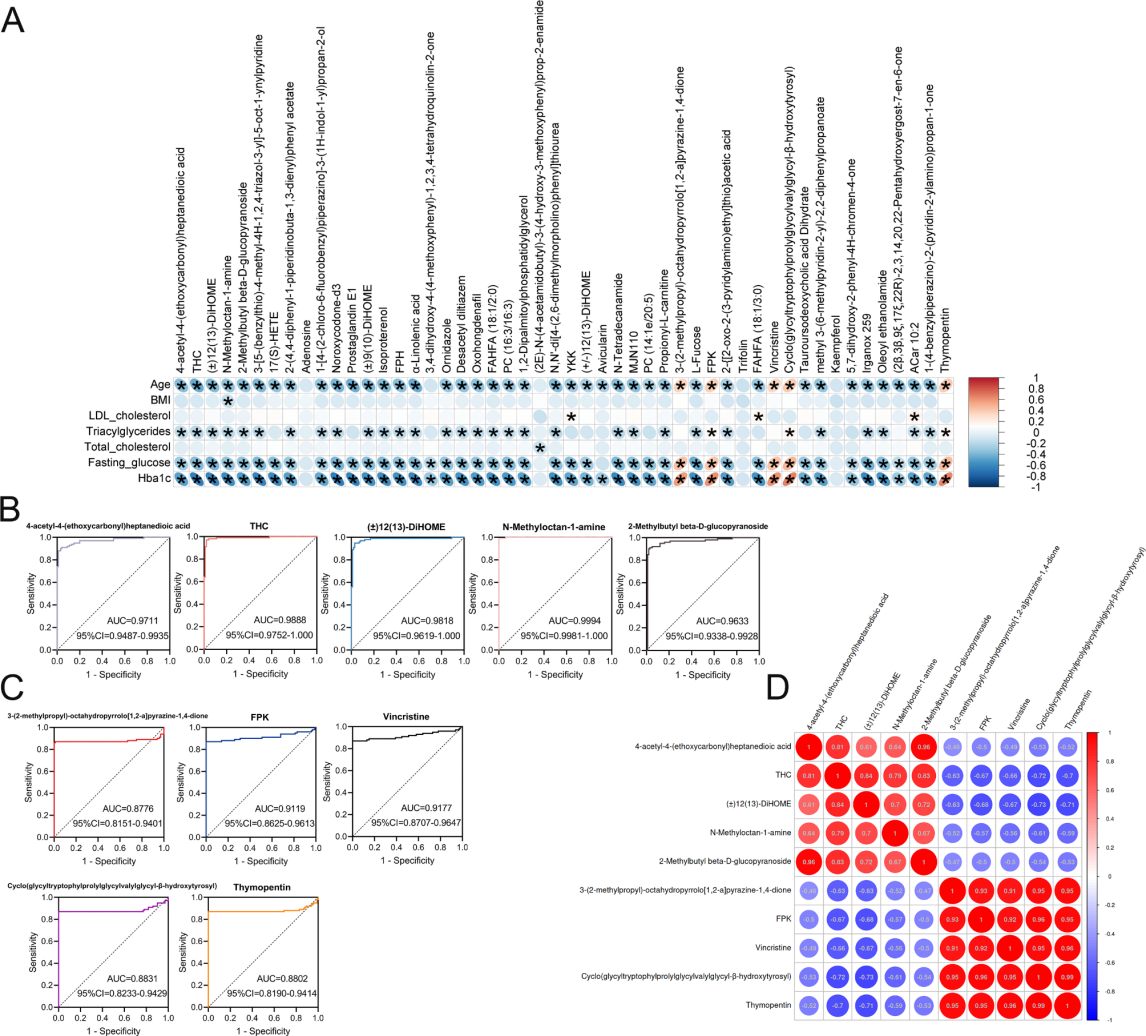
Potential biomarkers panel discovery for predicting T-T2DM. **(A)** A heat map shows the correlation between the top 50 metabolites of Gini impurity and clinical features (Red asterisks represent a positive correlation and blue asterisks represent a negative correlation). **(B)** ROC curves for each of the 5 selected potential biomarkers from the RFC model. **(C)** ROC curves for the five increased metabolites of the top 50 metabolites of Gini impurity. **(D)** The heat map illustrates Pearson correlations between potential biomarkers panel (Red circles, positive correlation; blue circles, negative correlation, the number inside each circle is Pearson correlation coefficient)

Given that the diagnostic value of serum metabolites for T-T2DM remains unknown, we systemically analyzed correlations between top 50 predictive metabolites of Gini importance and the clinical parameters of each patient (Supplementary Table 3). Impressively, most of the metabolites negatively correlated with age, triglycerides, FPG, and HbA1c, Meanwhile, four metabolites, namely, 3-(2-methylpropyl)-octahydropyrrolo[1,2-a]pyrazine-1,4-dione, FPK, vincristine, cyclo(glycyltryptophylprolylglycylvalylglycyl-β-hydroxytyrosyl) and thymopentin were significantly associated with increased T-T2DM risk (Fig. 5A). Furthermore, the predictive ability of T-T2DM was analyzed by ROC analysis. As shown in Fig. 5B, the top 5 predictive metabolites of Gini importance, especially THC, (±)12(13)-DiHOME and N-methyloctan-1-amine, had a stronger predictive power than FPG. These five metabolites were also identified as potentially predictive markers for T-T2DM (Fig. 5C**)**. We then assessed pairwise Pearson correlations between levels of these metabolites. The top 5 decreased metabolites and four increased metabolites showed strong mean correlations (r = 0.59, Fig. 5D**)**. Collectively, changes in serum metabolites mentioned above may be effective biomarkers for determining T-T2DM onset and progression.

## Discussion

Few studies have specifically reported the clinical characteristics of T2DM in Tibetan Chinese population. Hence, this retrospective cohort study described the detailed clinical features of Tibetans with T2DM in comparison with those of the healthy population. We found that the age, higher triglyceride, FPG, and HbA1c levels, and lower serum uric acid and creatinine levels were significant risk factors of T-T2DM. These risk factors were equally likely to develop long-term cardiovascular and kidney diseases [23]. The association of factors such as age [24], hypertriglyceridemia [25], and FPG [26],and HbA1c levels [27] with the risk of T2DM has been established. However, the association of the serum uric acid and creatinine levels with T2DM presence remains undetermined. Serum uric acid has been reported to be negatively associated with FPG, HbA1c, and high-density lipoprotein cholesterol [28, 29]. Other studies demonstrated lower serum uric acid level in the context of hyperglycemia, similar to our results [30, 31, 29], In addition, reduced serum creatinine levels were significantly associated with increased T2DM risk [32, 33]. This association could be explained by hyperfiltration of glomeruli and ectopic accumulation of adipose tissue combined with low muscle mass [34–36].

Physiological changes have been identified in Tibetans living at high altitudes, and many studies have unveiled the genetic bases of these physiological changes and a distinct metabolic signature for this population [37]. Tibetans may be vulnerable to glucose intolerance, with polycythemia as an indication of hypoxia adaptation [38]. Tibetans reportedly have higher oxidative stress than the Han counterparts, and a higher oxidative stress is associated with glucose intolerance and arteriosclerosis [39, 40]. The hypoxia-inducible factor pathway and metabolic features such as low cardiac phosphocreatine-to-ATP ratios, increased cardiac glucose uptake, and lower muscle mitochondrial densities have been observed in the high-altitude–adapted Tibetan native population [41]. These unique features contribute to Tibetans^’^ distinct metabolic changes.

To our knowledge, this study is the first to report novel predictive metabolic markers and altered metabolic profiles of T2DM among Tibetans in China. Significantly high levels of aromatic amino acids and BCAAs (leucine, isoleucine, and valine), low carbon number lipids (myristic, palmitic, and stearic acid), and significantly reduced pyroglutamic acid, glycerophospohlipids, and sphingomyelins are associated with T2DM [42, 43]. In the current study, the amino acids phenylalanine, tyrosine, and tryptophan were downregulated in the T-T2DM group compared with those in the HC group. Moreover, BCAA derivatives such as 4-hydroxyisoleucine (VIP value = 1.012, Q-value < 0.001, fold-change (T-T2DM/T-HC) = 0.689) and N-acetylvaline (VIP value = 1.145, Q-value < 0.001, fold-change (T-T2DM/T-HC) = 0.794) were downregulated in the T-T2DM group. In particular, 4-hydroxyisoleucine is useful for DM treatment because of its capacity for increasing insulin secretion [44]. Conversely, the levels of tripeptide compounds including QNK and FPK were increased in the T-T2DM group, and their increase was associated with DM risk. Further, β-hydroxybutyric acid (BHA) is a ketone body that has been described as an early biomarker of DM or diabetic ketoacidosis (DKA) [45, 46]. In DKA, increased free fatty acid oxidation and acidosis will lead to reduced mitochondrial redox state (nicotinamide adenine dinucleotide plus hydrogen-to-NAD1 ratio), promoting BHA production. Consistent with these results, the BHA level in our study (VIP value = 2.186, Q-value < 0.001, fold-change (T-T2DM/T-HC) = 1.499) was significantly higher in the T-T2DM group than in the T-HC group. Monitoring serum BHA levels may help early diagnose T-T2DM and detect DKA.

In network analyses, the metabolomic signatures were associated with phenylalanine metabolism; phenylalanine, tyrosine, and tryptophan biosynthesis; arachidonic acid metabolism, and riboflavin metabolism. Phenylalanine stimulates insulin secretion and further regulates compensatory mechanisms in the early stages of insulin resistance. Once compensated insulin secretion is met, individuals could subsequently progress to overt T2DM. Our results of metabolite changes in T-T2DM are consistent with recent findings in which decrease in aromatic amino acid levels occur after T2DM progression [47, 48]. In addition, the T-T2DM group had significantly reduced levels of free arachidonate and prostaglandin H2 in arachidonic acid metabolism; this significant decrement may also be a cause of glucose and lipid metabolism disorders in this group [49].

Several prospective metabolomic studies have investigated T2DM risks and biomarkers in a Chinese population by machine learning [48, 50]. A validated integrated biomarker profiling (IBP) was constructed using amino acids, L-carnitine, and acetyl-L-carnitine for the prediction of impaired fasting glucose and T2DM disease risks [50]. The present study used RFC to select the top 5 biomarkers of Gini impurity that have a good prediction ability of T-T2DM disease risk for IBP construction. These five biomarkers were 4-acetyl-4-(ethoxycarbonyl)heptanedioic acid, THC, (±)12(13)-DiHOME, N-methyloctan-1-amine, and 2-methylbutyl beta-D-glucopyranoside. The predicted performance of the model was satisfactory and better than the traditional markers of T2DM. These predictive metabolites negatively correlated with age, triglycerides, FPG, and HbA1c. Of note, (±)12(13)-DiHOME, an adipokine from brown adipose tissue, is closely related to the homeostasis of blood glucose and the metabolism balance of fatty acids and other lipids [51]. In addition, (±)12(13)-DiHOME is a peroxisome proliferator-activated receptor-γ receptor agonist that lowers blood glucose by enhancing systemic insulin sensitivity [52, 53]. However, (±)12(13)-DiHOME deletion in T-T2DM individuals could potentially contribute to a tightly linked interplay of increased oxidative stress and reduced insulin secretion, resulting in hyperglycemia secondary to inability to compensate for reduced insulin sensitivity.

This study has some limitations that should be considered. First, this study included patients from one institution only, and the sample size is small. Second, to capture a large number of metabolites, we used an untargeted metabolomic approach that could not measure the absolute values of metabolites. Nevertheless, this limitation did not impede our ability to estimate the associations between metabolites and the risk of T-T2DM. Third, some unmeasured factors (e.g., changes in lifestyle factors, or other diseased states over time) might have influenced our findings. Hence, our prospective study results should be interpreted with caution. Last, we only applied one machine learning method. More machine learning methods and further deep mining or algorithms may be needed. And our machine learning model was validated in the same cohort of subjects. We need to increase the sample volume for validating the results in an external cohort.

In conclusion, this study systematically profiled wide-ranging serum metabolites that were found to be associated with DM risk in Tibetan adults. Through metabolomics and a machine learning method, we have established the IBPs of T-T2DM and discovered potential biomarkers for predicting T-T2DM. Our findings may provide valuable diagnostic tools for the clinical implementation and design of effective novel therapeutic targets to achieve earlier T-T2DM prevention, diagnosis, and treatment.

## Electronic supplementary material

Below is the link to the electronic supplementary material.

Supplementary Material 1

## Data Availability

The raw data supporting the conclusions of this article will be made available by the authors, without undue reservation, to any qualified researcher.

## Abbreviations

T-T2DM: Tibetan with type 2 diabetes mellitus
DM: Diabetes mellitus
T2DM: Type 2 diabetes mellitus
FPG: Fasting plasma glucose
BMI: body mass index
LC-MS: liquid chromatography mass spectrometry
SBP: Systolic blood pressure
DBP: Diastolic blood pressure
HbA1c: Hemoglobin A1c, LDL:Low-density lipoprotein
HDL: High-density lipoprotein
OGTT: Oral glucose tolerance test
T-HC: Tibetan healthy controls
ROC: Receiver operating characteristic
AUC: Area under the curve
IBP: Integrated biomarker profiling
