# Supplementary material for: Integrated biomarker profiling of the metabolome associated with type 2 diabetes mellitus among Tibetan in China
Source: Diabetol Metab Syndr. 2023 Jul 1;15:146. doi: 10.1186/s13098-023-01124-8 (PMC10314538; doi:10.1186/s13098-023-01124-8)
Supplement: Supplementary file 1 — Supplementary Material 1 [file 13098_2023_1124_MOESM1_ESM.docx]

**Supplementary Methods and Materials for**

**Integrated biomarker profiling of the metabolome associated with type 2 diabetes mellitus among Tibetan in China**

**Supplementary Methods**

**Supplementary Table 1**. Significant metabolites between T-T2DM group (n=100) and T-HC group (n=100) in serum.

**Supplementary Table 2.** Significant functional KEGG pathways involved according to the differentially expressed metabolites**.**

**Supplementary Table 3.** The top 50 metabolites of of Gini impurity in Random Forest model.

**Supplementary Figure 1.** Paired differences in metabolite concentration between T-T2DM group (n=100) and T-HC group (n=100).

***Metabolites preparation and extraction***

The collected serum samples were thawed on ice. Briefly, 100 µL of plasma were mixed with 400µL of 80% ice-cold methanol by well vortex. Then the samples were incubated on ice for 5 min and centrifuged at 15,000 g, 4°C for 20 min. Some of supernatant was diluted to final concentration containing 53% methanol by LC-MS grade water. The samples were centrifuged at 15000 g, for 20 min at 4°C. Finally, the supernatant was injected into the LC-MS/MS system analysis. Quality control (QC) samples were prepared by pooling aliquots of all the serum samples and were processed with the same procedure used for the experimental samples. During analysis of the samples, one quality control sample was run after every 10 injections.

***Ultra high performance liquid chromatography–mass spectrometry method for metabolomics***

UHPLC-MS/MS analyses were performed using a Vanquish UHPLC system (ThermoFisher, Germany) coupled with an Orbitrap Q Exactive^TM^ HF-X mass spectrometer (Thermo Fisher，Germany) in Novogene Co., Ltd. (Beijing, China) via a previously described method[1, 2]. The processed samples were injected onto a Hypesil Gold column (100×2.1 mm, 1.9μm) using a 17-min linear gradient at a flow rate of 0.2mL/min. For the positive polarity mode, the eluents were eluent A (0.1% FA in Water) and eluent B (Methanol), for the negative polarity mode, eluent A (5 mMammonium acetate, pH 9.0) and eluent B (Methanol), respectively. The solvent gradient was set as follows: 2% B, 1.5 min; 2-100% B, 3 min; 100% B, 10 min; 100-2% B, 10.1 min; 2% B, 12 min. Q Exactive^TM^ HF-X mass spectrometer was operated in positive/negative polarity mode with spray voltage of 3.5 kV, capillary temperature of 320°C, sheath gas flow rate of 35 psi and aux gas flow rate of 10 L/min, S-lens RF level of 60, Aux gas heater temperature of 350°C.

***Data processing and metabolite identification***

The raw data files generated by UHPLC-MS/MS were processed using the Compound Discoverer 3.1 (CD3.1, ThermoFisher) to perform peak alignment, peak picking, and quantitation for each metabolite. The main parameters were set as follows: retention time tolerance, 0.2 minutes; actual mass tolerance, 5ppm; signal intensity tolerance, 30%; signal/noise ratio, 3; and minimum intensity, et al. After that, peak intensities were normalized to the total spectral intensity. The data normalization step was completed by MetaboAnalyst 5.0 (<https://www.metaboanalyst.ca/>). We reduced the resulting matrix by replacing all the missing values with a small value and predicted the molecular formula based on additive ions, molecular ion peaks and fragment ions. And then peaks were matched with the mzCloud (<https://www.mzcloud.org/>), mzVault and MassList database to obtain the accurate qualitative and relative quantitative results. Additional statistical analysis using univariate and multivariate statistical techniques was performed on the identified lipids.

***Machine learning analysis***

We used the validation phase dataset including 200 samples to establish a model to predict the risk of T-T2DM. The dataset was segmented as the training set (67 healthy subjects, 73 with T-T2DM) and test set (33 healthy subjects, 27 with T-T2DM) using the 70−30 holdout method. The models were trained with the training set to optimize the parameters. Then, the test set was used to evaluate the performance of the model. The concentrations of the potential biomarkers were set as covariates and disease status was set as the dependent variable. The model was trained using the training set.

LR was a classic algorithm to evaluate the probability of features associated with a disease, which could be used for dichotomy and multi-classification problems. The cross-validation error curves from the 5 trials were averaged, and the minimum error in the averaged curve plus the standard deviation at that point was identified as the cutoff for an acceptable error by the LR algorithm.

To improve the sensitivity of the IBP prediction model for T-T2DM, analysis of variance, Mean Decrease in Accuracy, and Gini impurity were used to rank potential biomarkers by importance. The higher the Gini impurity, the more important the potential biomarker. The ROC curve was used to evaluate the predictive performance of the current method across the entire range of algorithm decision values. The ROC curve was illustrated with the true positive rate (TPR) and false positive rate (FPR), which could reveal the relationship between sensitivity and specificity. The Receiver operating characteristic (ROC) curves and area under the curve (AUC) were calculated by R package ‘pROC’.

**REFERENCES**

1. Dunn WB, Broadhurst D, Begley P *et al*: Procedures for large-scale metabolic profiling of serum and plasma using gas chromatography and liquid chromatography coupled to mass spectrometry. *Nat Protoc* 2011, 6(7):1060-1083.(2011)

2. Fan KQ, Li YY, Wang HL *et al*: Stress-Induced Metabolic Disorder in Peripheral CD4(+) T Cells Leads to Anxiety-like Behavior. *Cell* 2019, 179(4):864-879 e819.(2019)

**Table S1.** Significant metabolites between T-T2DM group (n=100) and T-HC group (n=100) in serum.

| Compound ID | Name | RT^a^ (min) | *m/z* | VIP | *P*-value^b^ | Fold change (T2DM/HC) | Trend^c^ |
| --- | --- | --- | --- | --- | --- | --- | --- |
| Com_678_pos | QNK | 5.912 | 389.215 | 2.634 | 1.20E-44 | 858.194 | ↑ |
| Com_7180_pos | FPK | 5.858 | 391.231 | 2.663 | 6.14E-45 | 60.658 | ↑ |
| Com_5047_pos | Cyclo(glycyltryptophylprolylglycylvalylglycyl-β-hydroxytyrosyl) | 10.187 | 715.320 | 2.572 | 2.65E-41 | 50.054 | ↑ |
| Com_3191_pos | Thymopentin | 9.458 | 680.373 | 2.601 | 1.05E-40 | 41.866 | ↑ |
| Com_8823_pos | 6beta-Naltrexol-d3 | 5.839 | 347.205 | 2.648 | 5.50E-46 | 33.821 | ↑ |
| Com_15119_pos | Vincristine | 8.551 | 847.379 | 2.629 | 2.21E-43 | 21.593 | ↑ |
| Com_374_pos | Phlorizin | 5.64 | 459.127 | 1.668 | 8.48E-19 | 5.315 | ↑ |
| Com_13658_pos | 3-(2-methylpropyl)-octahydropyrrolo[1,2-a]pyrazine-1,4-dione | 9.486 | 233.121 | 2.482 | 1.09E-31 | 3.584 | ↑ |
| Com_7153_pos | Inosine | 3.267 | 269.088 | 1.498 | 1.18E-13 | 3.416 | ↑ |
| Com_237_pos | Hypoxanthine | 1.678 | 137.046 | 1.500 | 5.04E-14 | 2.042 | ↑ |
| Com_18166_pos | Limonin | 6.51 | 453.189 | 1.577 | 2.72E-11 | 1.984 | ↑ |
| Com_12227_neg | 4-(2-thienyl)benzoic acid | 1.737 | 203.019 | 1.967 | 2.19E-19 | 1.817 | ↑ |
| Com_3036_neg | 3,4-Dihydroxyphenylpropionic acid | 5.379 | 181.051 | 1.244 | 0.000598074 | 0.833 | ↓ |
| Com_27493_neg | 7-(trifluoromethyl)thieno[3,2-b][1,5]benzoxazepin-10(9H)-one | 4.902 | 284.001 | 1.214 | 1.15E-06 | 0.832 | ↓ |
| Com_11587_neg | 5-oxoproline | 1.7 | 128.036 | 1.468 | 0.000244438 | 0.832 | ↓ |
| Com_3152_neg | Citraconic acid | 2.012 | 129.020 | 1.497 | 0.000415179 | 0.832 | ↓ |
| Com_3628_neg | Pantothenic acid | 5.075 | 218.104 | 1.119 | 0.025467285 | 0.832 | ↓ |
| Com_20405_pos | N-[1-(4-methoxy-2-oxo-2H-pyran-6-yl)-2-methylbutyl]acetamide | 5.018 | 254.139 | 1.034 | 3.22E-09 | 0.830 | ↓ |
| Com_10257_neg | 2-Isopropylmalate | 5.771 | 175.061 | 1.185 | 0.000151038 | 0.829 | ↓ |
| Com_13373_neg | Sebacic acid | 6.337 | 201.114 | 1.015 | 6.46E-05 | 0.829 | ↓ |
| Com_272_neg | Indole-3-lactic acid | 5.674 | 204.067 | 1.001 | 0.001984464 | 0.829 | ↓ |
| Com_19828_neg | Cyclocytidine | 5.181 | 260.045 | 1.187 | 3.09E-07 | 0.829 | ↓ |
| Com_2063_pos | 2,4-Dimethylbenzaldehyde | 6.778 | 135.081 | 1.585 | 2.29E-16 | 0.822 | ↓ |
| Com_7443_neg | N1-(4-morpholinophenyl)-2-methylbut-2-enamide | 5.809 | 259.145 | 1.008 | 8.85E-06 | 0.821 | ↓ |

^a^ Retention time of the metabolites during LC-MS. ^b^ *P* values were calculated by Student’s t-test (threshold<0.05). ^c^ Trend, the up and down arrows represent a relative increase and decrease, respectively, in the level of the metabolite in the T-T2DM group when compared with T-HC group.

**Table S2.** Significant functional KEGG pathways involved according to the differentially expressed metabolites**.**

| MapID | MapTitle | Pvalue | -LOG(p,e) | Impact | MetaIDs |
| --- | --- | --- | --- | --- | --- |
| hsa00360 | Phenylalanine metabolism | 0.93125 | 0.07123 | 0.77777 | L-Phenylalanine;Phenethylamine;Phenylpyruvate;2-Hydroxyphenylacetate |
| hsa00400 | Phenylalanine, tyrosine and tryptophan biosynthesis | 0.84172 | 0.17231 | 0.75 | Phenylpyruvate;L-Phenylalanine |
| hsa00590 | Arachidonic acid metabolism | 0.84172 | 0.17231 | 0.47222 | Arachidonate;Prostaglandin H2 |
| hsa00740 | Riboflavin metabolism | 0.93706 | 0.06501 | 0.4 | Riboflavin |
| hsa00290 | Valine, leucine and isoleucine biosynthesis | 0.93706 | 0.06501 | 0.25 | L-Threonine |
| hsa00380 | Tryptophan metabolism | 0.99901 | 0.00099 | 0.23076 | Serotonin;3-Hydroxyanthranilate;L-Kynurenine |
| hsa00072 | Synthesis and degradation of ketone bodies | 0.74638 | 0.29252 | 0.14286 | Acetoacetate |
| hsa00140 | Steroid hormone biosynthesis | 1 | ####### | 0.13131 | Pregnenolone;17alpha-Hydroxypregnenolone;11-Deoxycorticosterone;Corticosterone;Androsterone;Androsterone glucuronide |
| hsa00592 | alpha-Linolenic acid metabolism | 0.74638 | 0.29252 | 0.125 | (9Z,12Z,15Z)-Octadecatrienoic acid |
| hsa00220 | Arginine biosynthesis | 0.99592 | 0.00409 | 0.125 | L-Citrulline;L-Ornithine |
| hsa00230 | Purine metabolism | 0.99227 | 0.00776 | 0.11629 | Xanthine;Adenosine;Hypoxanthine;Inosine;Deoxyguanosine;Guanosine;Urate |
| hsa00330 | Arginine and proline metabolism | 0.73366 | 0.3097 | 0.1 | Spermidine;cis-4-Hydroxy-D-proline;L-Ornithine |
| hsa00240 | Pyrimidine metabolism | 0.96391 | 0.03676 | 0.08475 | Uridine;Thymidine;Orotate |
| hsa00520 | Amino sugar and nucleotide sugar metabolism | 0.94977 | 0.05154 | 0.07692 | D-Glucosamine 6-phosphate;6-Deoxy-L-galactose |
| hsa00970 | Aminoacyl-tRNA biosynthesis | 0.99973 | 0.00027 | 0.06896 | L-Phenylalanine;L-Threonine |
| hsa00650 | Butanoate metabolism | 0.93706 | 0.06501 | 0.06667 | Acetoacetate |
| hsa00270 | Cysteine and methionine metabolism | 0.84172 | 0.17231 | 0.0606 | S-Adenosyl-L-homocysteine;L-Cystine |
| hsa00770 | Pantothenate and CoA biosynthesis | 0.98473 | 0.01539 | 0.05556 | Pantothenate |
| hsa00480 | Glutathione metabolism | 0.84172 | 0.17231 | 0.05406 | L-Ornithine;Spermidine |
| hsa00900 | Terpenoid backbone biosynthesis | 0.74638 | 0.29252 | 0.05 | (R)-Mevalonate |

**Table S3.** The top 50 metabolites of of Gini impurity in Random Forest model.

| Compound_ID | MeanDecreaseGini | Name | Formula | m/z | RT [min] | FC(T-T2DM/THC) | log2FC | Pvalue | VIP | Up.Down |
| --- | --- | --- | --- | --- | --- | --- | --- | --- | --- | --- |
| Com_5601_pos | 3.240 | 4-acetyl-4-(ethoxycarbonyl)heptanedioic acid | C12 H18 O7 | 275.11 | 5.16 | 0.137 | -2.869 | 2.81E-51 | 2.442 | down |
| Com_18581_neg | 3.232 | THC | C21 H30 O2 | 313.221 | 10.06 | 0.133 | -2.909 | 4.50E-72 | 2.709 | down |
| Com_1224_neg | 2.860 | (±)12(13)-DiHOME | C18 H34 O4 | 313.239 | 7.155 | 0.092 | -3.447 | 1.49E-55 | 2.549 | down |
| Com_1145_pos | 2.820 | N-Methyloctan-1-amine | C9 H21 N | 144.175 | 5.007 | 0.005 | -7.526 | 6.54E-122 | 2.874 | down |
| Com_6072_neg | 2.719 | 2-Methylbutyl beta-D-glucopyranoside | C11 H22 O6 | 295.14 | 5.268 | 0.193 | -2.375 | 3.49E-43 | 2.252 | down |
| Com_8658_pos | 2.420 | 3-[5-(benzylthio)-4-methyl-4H-1,2,4-triazol-3-yl]-5-oct-1-ynylpyridine | C23 H26 N4 S | 391.195 | 5.588 | 0.119 | -3.072 | 8.27E-57 | 2.611 | down |
| Com_1255_pos | 2.312 | 17(S)-HETE | C20 H32 O3 | 321.241 | 8.292 | 0.328 | -1.610 | 2.89E-40 | 2.225 | down |
| Com_3092_pos | 2.247 | 2-(4,4-diphenyl-1-piperidinobuta-1,3-dienyl)phenyl acetate | C29 H29 N O2 | 406.219 | 5.611 | 0.071 | -3.810 | 5.51E-59 | 2.596 | down |
| Com_9913_pos | 2.217 | Adenosine | C10 H13 N5 O4 | 268.108 | 5.637 | 0.770 | -0.378 | 1.11E-34 | 2.308 | down |
| Com_15264_pos | 1.760 | 1-[4-(2-chloro-6-fluorobenzyl)piperazino]-3-(1H-indol-1-yl)propan-2-ol | C22 H25 Cl F N3 O | 402.174 | 5.546 | 0.456 | -1.132 | 1.79E-43 | 2.150 | down |
| Com_3498_pos | 1.752 | Noroxycodone-d3 | C17 H16 [2]H3 N O4 | 305.158 | 5.669 | 0.152 | -2.720 | 3.76E-55 | 2.595 | down |
| Com_3429_pos | 1.747 | Prostaglandin E1 | C20 H34 O5 | 337.235 | 7.231 | 0.213 | -2.234 | 6.42E-47 | 2.440 | down |
| Com_1375_neg | 1.680 | (±)9(10)-DiHOME | C18 H34 O4 | 313.239 | 7.315 | 0.233 | -2.100 | 2.46E-48 | 2.389 | down |
| Com_7242_pos | 1.624 | Isoproterenol | C11 H17 N O3 | 212.128 | 5.664 | 0.436 | -1.199 | 6.85E-51 | 2.275 | down |
| Com_2389_pos | 1.481 | FPH | C20 H25 N5 O4 | 382.19 | 5.544 | 0.073 | -3.779 | 2.19E-55 | 2.617 | down |
| Com_2188_pos | 1.408 | α-Linolenic acid | C18 H30 O2 | 279.233 | 6.969 | 0.176 | -2.507 | 8.60E-65 | 2.562 | down |
| Com_20707_pos | 1.285 | 3,4-dihydroxy-4-(4-methoxyphenyl)-1,2,3,4-tetrahydroquinolin-2-one | C16 H15 N O4 | 286.108 | 5.722 | 0.343 | -1.542 | 3.53E-40 | 2.338 | down |
| Com_5200_pos | 1.283 | Ornidazole | C7 H10 Cl N3 O3 | 220.049 | 5.524 | 0.495 | -1.015 | 2.00E-41 | 1.888 | down |
| Com_8145_pos | 1.171 | Desacetyl diltiazem | C20 H24 N2 O3 S | 373.159 | 5.527 | 0.500 | -1.000 | 7.73E-45 | 1.949 | down |
| Com_625_pos | 1.163 | Oxohongdenafil | C25 H32 N6 O4 | 481.263 | 5.438 | 0.359 | -1.479 | 1.92E-23 | 1.984 | down |
| Com_16188_neg | 1.160 | FAHFA (18:1/2:0) | C20 H36 O4 | 339.254 | 11.13 | 0.141 | -2.829 | 1.34E-56 | 2.482 | down |
| Com_9994_pos | 1.136 | PC (16:3/16:3) | C40 H68 N O8 P | 722.471 | 9.725 | 0.211 | -2.244 | 1.15E-48 | 2.445 | down |
| Com_5274_pos | 1.079 | 1,2-Dipalmitoylphosphatidylglycerol | C38 H75 O10 P | 745.509 | 10.26 | 0.172 | -2.544 | 2.31E-47 | 2.529 | down |
| Com_16812_pos | 1.053 | (2E)-N-(4-acetamidobutyl)-3-(4-hydroxy-3-methoxyphenyl)prop-2-enamide | C16 H22 N2 O4 | 329.144 | 5.535 | 0.760 | -0.396 | 7.24E-23 | 1.957 | down |
| Com_1382_pos | 1.003 | N,N'-di[4-(2,6-dimethylmorpholino)phenyl]thiourea | C25 H34 N4 O2 S | 477.232 | 5.375 | 0.074 | -3.765 | 1.13E-54 | 2.594 | down |
| Com_4498_pos | 0.944 | YKK | C21 H35 N5 O5 | 875.527 | 9.629 | 0.218 | -2.199 | 7.65E-43 | 2.426 | down |
| Com_1816_neg | 0.904 | (+/-)12(13)-DiHOME | C18 H34 O4 | 295.228 | 8.105 | 0.323 | -1.630 | 8.33E-40 | 2.185 | down |
| Com_3898_pos | 0.895 | Avicularin | C20 H18 O11 | 435.093 | 5.612 | 0.414 | -1.273 | 4.52E-38 | 2.140 | down |
| Com_4350_pos | 0.894 | N-Tetradecanamide | C14 H29 N O | 228.233 | 9.684 | 0.537 | -0.896 | 1.70E-50 | 2.311 | down |
| Com_13194_pos | 0.845 | MJN110 | C22 H21 Cl2 N3 O4 | 462.102 | 5.744 | 0.419 | -1.254 | 2.03E-48 | 2.283 | down |
| Com_14358_pos | 0.815 | PC (14:1e/20:5) | C42 H74 N O7 P | 736.522 | 10.2 | 0.288 | -1.798 | 1.87E-42 | 2.351 | down |
| Com_21779_neg | 0.813 | Propionyl-L-carnitine | C10 H19 N O4 | 216.124 | 6.39 | 0.229 | -2.125 | 8.09E-43 | 2.262 | down |
| Com_13658_pos | 0.714 | 3-(2-methylpropyl)-octahydropyrrolo[1,2-a]pyrazine-1,4-dione | C11 H18 N2 O2 | 233.121 | 9.486 | 3.584 | 1.842 | 1.09E-31 | 2.482 | up |
| Com_223_neg | 0.569 | L-Fucose | C6 H12 O5 | 199.038 | 1.405 | 0.173 | -2.534 | 9.89E-40 | 2.404 | down |
| Com_7180_pos | 0.567 | FPK | C20 H30 N4 O4 | 391.231 | 5.858 | ##### | 5.923 | 6.14E-45 | 2.663 | up |
| Com_15072_pos | 0.529 | 2-{[2-oxo-2-(3-pyridylamino)ethyl]thio}acetic acid | C9 H10 N2 O3 S | 249.029 | 1.39 | 0.337 | -1.571 | 6.14E-45 | 2.369 | down |
| Com_4018_pos | 0.506 | Trifolin | C21 H20 O11 | 449.109 | 5.65 | 0.214 | -2.225 | 8.05E-33 | 1.928 | down |
| Com_22143_neg | 0.500 | FAHFA (18:1/3:0) | C21 H38 O4 | 353.27 | 11.07 | 0.169 | -2.568 | 2.86E-49 | 2.487 | down |
| Com_15119_pos | 0.484 | Vincristine | C46 H56 N4 O10 | 847.379 | 8.551 | ##### | 4.433 | 2.21E-43 | 2.629 | up |
| Com_5047_pos | 0.478 | Cyclo(glycyltryptophylprolylglycylvalylglycyl-β-hydroxytyrosyl) | C36 H44 N8 O9 | 715.32 | 10.19 | ##### | 5.645 | 2.65E-41 | 2.572 | up |
| Com_9714_pos | 0.426 | Tauroursodeoxycholic acid Dihydrate | C26 H49 N O8 S | 536.325 | 10.09 | 0.260 | -1.942 | 8.57E-39 | 2.309 | down |
| Com_16032_pos | 0.426 | methyl 3-(6-methylpyridin-2-yl)-2,2-diphenylpropanoate | C22 H21 N O2 | 332.167 | 5.521 | 0.523 | -0.935 | 1.18E-40 | 1.876 | down |
| Com_1303_pos | 0.423 | Kaempferol | C27 H30 O15 | 595.167 | 5.41 | 0.349 | -1.521 | 7.91E-12 | 1.456 | down |
| Com_18386_pos | 0.410 | 5,7-dihydroxy-2-phenyl-4H-chromen-4-one | C15 H10 O4 | 255.065 | 5.515 | 0.508 | -0.978 | 1.30E-22 | 1.689 | down |
| Com_6097_pos | 0.396 | Irganox 259 | C40 H62 O6 | 677.409 | 9.748 | 0.143 | -2.807 | 6.34E-55 | 2.529 | down |
| Com_521_pos | 0.362 | Oleoyl ethanolamide | C20 H39 N O2 | 326.306 | 9.696 | 0.257 | -1.960 | 6.66E-43 | 2.441 | down |
| Com_172_pos | 0.339 | (2β,3β,9ξ,17ξ,22R)-2,3,14,20,22-Pentahydroxyergost-7-en-6-one | C28 H46 O6 | 497.343 | 9.06 | 0.710 | -0.494 | 1.18E-13 | 1.375 | down |
| Com_2035_pos | 0.322 | ACar 10:2 | C17 H30 N O4 | 312.218 | 5.761 | 0.402 | -1.314 | 1.59E-34 | 2.016 | down |
| Com_8588_pos | 0.262 | 1-(4-benzylpiperazino)-2-(pyridin-2-ylamino)propan-1-one | C19 H24 N4 O | 363.164 | 5.31 | 0.255 | -1.971 | 7.20E-31 | 2.091 | down |
| Com_3191_pos | 0.260 | Thymopentin | C30 H49 N9 O9 | 680.373 | 9.458 | ##### | 5.388 | 1.05E-40 | 2.601 | up |

Note: RT [min], Retention time of the metabolites during LC-MS. FC, fold-changes; *P* values were calculated by Student’s t-test (threshold<0.05); VIP, variable importance in projection.

**Figure S1.** Paired differences in metabolite concentration between T-T2DM group (n=100) and T-HC group (n=100).


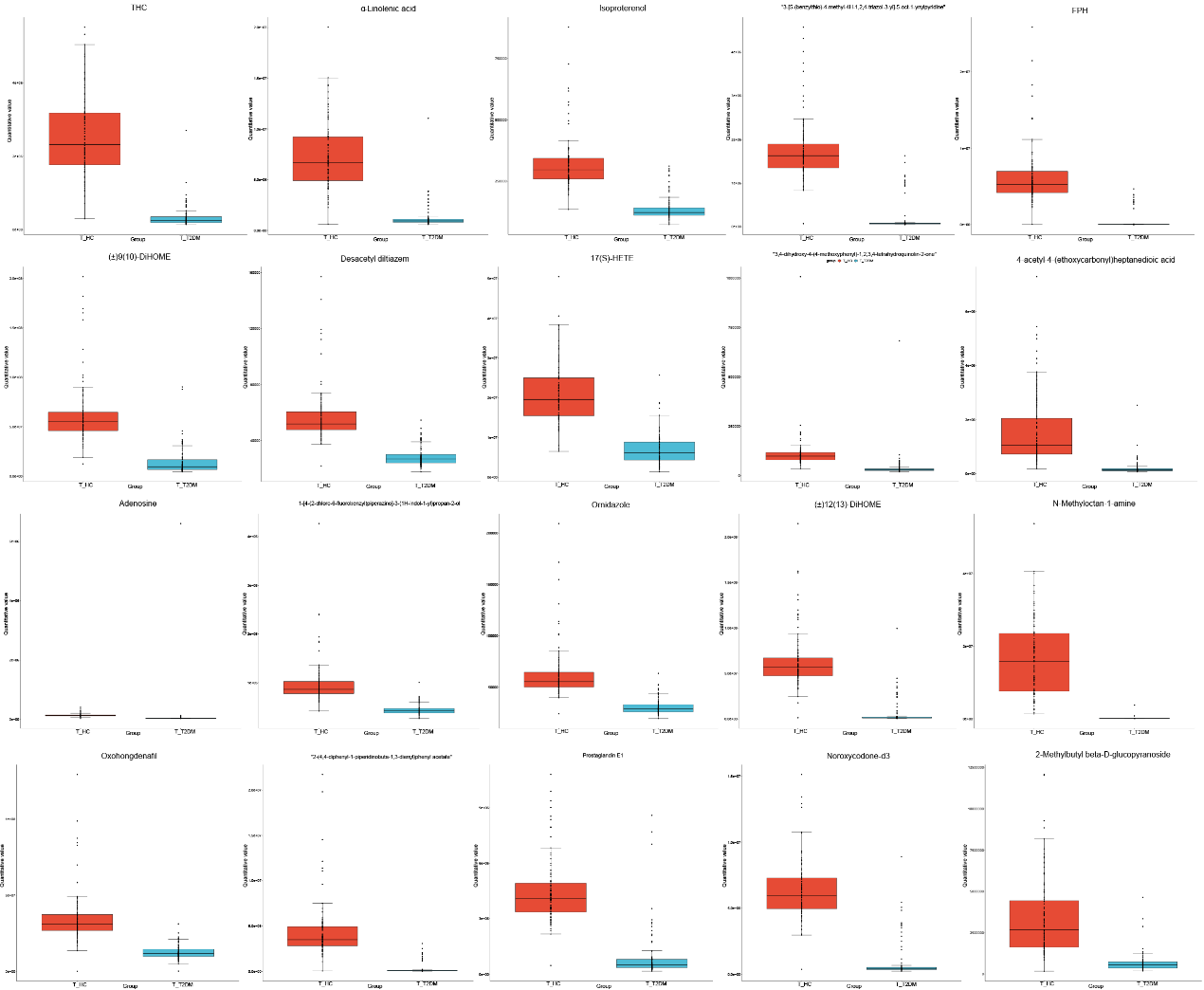


Note: T-T2DM, Tibetan type 2 diabetes mellitus; T-HC, Tibetan healthy controls.
